# Genetic Factors Are Not the Major Causes of Chronic Diseases

**DOI:** 10.1371/journal.pone.0154387

**Published:** 2016-04-22

**Authors:** Stephen M. Rappaport

**Affiliations:** School of Public Health, University of California, Berkeley, California, United States of America; University of Newcastle, AUSTRALIA

## Abstract

The risk of acquiring a chronic disease is influenced by a person’s genetics (G) and exposures received during life (the ‘exposome’, E) plus their interactions (G×E). Yet, investigators use genome-wide association studies (GWAS) to characterize G while relying on self-reported information to classify E. If E and G×E dominate disease risks, this imbalance obscures important causal factors. To estimate proportions of disease risk attributable to G (plus shared exposures), published data from Western European monozygotic (MZ) twins were used to estimate population attributable fractions (PAFs) for 28 chronic diseases. Genetic PAFs ranged from 3.4% for leukemia to 48.6% for asthma with a median value of 18.5%. Cancers had the lowest PAFs (median = 8.26%) while neurological (median = 26.1%) and lung (median = 33.6%) diseases had the highest PAFs. These PAFs were then linked with Western European mortality statistics to estimate deaths attributable to G for heart disease and nine cancer types. Of 1.53 million Western European deaths in 2000, 0.25 million (16.4%) could be attributed to genetics plus shared exposures. Given the modest influences of G-related factors on the risks of chronic diseases in MZ twins, the disparity in coverage of G and E in etiological research is problematic. To discover causes of disease, GWAS should be complemented with exposome-wide association studies (EWAS) that profile chemicals in biospecimens from incident disease cases and matched controls.

## Introduction

As the world’s population ages, mortality increasingly reflects the ravages of complex chronic diseases, particularly cancer and heart disease [1]. A person’s risk of succumbing to a chronic disease is linked to his or her genetics (G) and exposome (E, representing all exposures during life) plus G×E interactions. Although geneticists and epidemiologists have debated the importance of G and E as causes of chronic diseases, it is clear that both factors affect disease risks [2, 3]. However, most etiologic research has focused on genetic causes and has relegated exposures to secondary roles. For example, when queried on Feb. 6, 2016, there were 566,685 PubMed citations for the keywords “disease causes AND genetics” compared to 71,922 citations for “disease causes AND exposure”, a ratio of about eight to one.

This genome-centric view of causation is motivated by the technologic ability to detect and manipulate genes, and fosters the notion that genetic factors are necessary determinants of disease that operate in a causal background of diverse exposures [4, 5]. Certainly, technologies spawned by the human genome project led to stunningly comprehensive genome-wide association studies (GWAS) that investigated genomic variability across thousands of diseased and healthy subjects. Yet, because more than 2,000 GWAS rarely reported relative risks greater than 1.2 [6, 7], geneticists are turning to whole-genome sequencing in searches for ‘missing heritability’ [8, 9]. This motivation stems, at least in part, from calculations of heritability that do not differentiate disease variation arising from genetic factors and shared exposures [10].

In contrast to GWAS, the epidemiology of causal exposures still relies on self-reported and geographic information plus a few targeted measurements [11, 12], much as it did a century ago. Nonetheless, data from the World Health Organization (WHO) has attributed nearly half of global mortality to a handful of exposures (Table 1), mainly particulate air pollution (including indoor smoke and occupational exposure) (14% of all deaths), tobacco smoking and second-hand smoke (13%), high plasma levels of sodium (6%), and alcohol use (which is generally protective but can be harmful with high consumption) (5%) [13]. There is also strong epidemiologic evidence that genetically-stable populations experience profound alterations in cancer incidence across generations and with migration that logically reflect changing exposures [3, 14, 15]. Thus, the empirical evidence promotes the notion that exposures are necessary determinants of disease that operate in a causal background of genetic diversity. However, compared to GWAS, the universe of exposures that has been investigated for associations with chronic diseases essentially consists of airborne particulate matter plus a set of about 300 environmental chemicals and nutrients [16].

**Table 1:** Global deaths attributed to exposure-risk factors for chronic diseases.

Risk factor	Attributed deaths	Percent of global deaths
Tobacco smoking	5,695,349	11.28
Indoor smoke	3,478,773	6.89
Ambient particulate pollution	3,223,540	6.38
Diet high in sodium	3,104,308	6.15
Alcohol use	2,735,511	5.42
Diet low in seafood omega-3 fatty acids	1,389,896	2.75
Lead exposure	674,038	1.33
Second-hand smoke	601,938	1.19
Diet low in polyunsaturated fatty acids	533,603	1.06
Diet high in trans fatty acids	515,260	1.02
Occupational chemicals	373,738	0.74
Drug use	157,805	0.31
Ambient ozone pollution	152,434	0.30
Diet low in calcium	125,594	0.25
Vitamin A deficiency	119,762	0.24
Iron deficiency	119,608	0.24
Residential radon	98,992	0.20
Zinc deficiency	97,330	0.19
**TOTAL**	**23,197,479**	**45.9**

Data were obtained from World Health Organization estimates of exposures affecting 50,506,784 global deaths in 2010 [13]. (Because of possible correlations across risk factors, attributed deaths and percentages may overestimate true values).

To investigate the global influence of genetic factors on chronic-disease risks, data from cohorts of monozygotic (MZ) twins in Western Europe were compiled to estimate population attributable fractions (PAFs) for 28 chronic diseases, including prominent cancers, cardiovascular diseases, neurologic diseases, lung diseases, and autoimmune diseases. Because pairs of MZ twins have essentially identical genomes and also share many exposures [10], especially in early life, these PAFs estimate proportions of cases that would *theoretically* be prevented if interventions were able to remove particular combinations of genotypes and shared exposures [17, 18]. To further evaluate the impacts of G and E on the risks of chronic diseases, PAFs from MZ twins were linked with mortality statistics from Western Europe to estimate the numbers of deaths attributable to genetic factors and shared exposures for ischemic heart disease and prominent cancers.

## Materials and Methods

Data for estimation of PAFs were obtained from publications of disease phenotypes in large MZ-twin cohorts, i.e. [19–35], some of which had been curated by Roberts *et al*. [36]. Virtually all of the data were from Western European twins, primarily Swedish, Danish, and Finnish. The 28 diseases included nine types of cancer, cardiovascular diseases (heart disease and stroke), neurological diseases (Parkinson’s disease, Alzheimer’s disease, dementia, and migraine), lung diseases (chronic obstructive pulmonary disease and asthma), obesity-associated diseases (Type-2 diabetes and gallstone disease), autoimmune diseases (rheumatoid arthritis, Type-1 diabetes, and thyroid autoimmunity), genitourinary diseases (general dystocia, stress urinary incontinence, and pelvic organ prolapse), and three other syndromes (chronic fatigue, irritable bowel syndrome, and gastroesophageal reflux). The following data were extracted from each study: gender, number of MZ twin pairs (*N*_T_), number of concordant MZ pairs (*N*_C_), and number of discordant MZ pairs (*N*_D_). Each PAF (%) was estimated as P*(RR-1)/RR, where P = 2*N*_C_/(2*N*_C_+*N*_D_)*100 represents the proportion (%) of case twins with an affected co-twin and RR = [2*N*_C_/(2*N*_C_+*N*_D_)]/[*N*_D_ /(*N*_D_+2*N*_T_-2(*N*_C_+*N*_D_)] is the associated relative risk. If statistics were reported for both male and female twin pairs, then PAFs were estimated for the combined datasets. Since all of the studies were reported around the year 2000 for twins from Western Europe, mortality statistics for Western Europeans in that year were obtained for ischemic heart disease and relevant cancers from the WHO Global Burden of Disease Database [37, 38]. To estimate deaths attributable to genetics and shared exposures, the number of deaths for each disease type were multiplied by the corresponding PAF from MZ twins.

## Results and Discussion

Statistics from studies of MZ twins are summarized in Table 2. Estimated G-related PAFs ranged from 3.4% for leukemia to 48.6% for asthma with a median value of 18.5% and interquartile range of 9.9% to 24.2%. This indicates that fractions of cases attributable to genetics plus shared exposures tend to be modest, with three fourths of the phenotypes having PAFs less than 25%. In fact, G-related PAFs for only two phenotypes were greater than 40%, i.e. thyroid autoimmunity (42%) and asthma (49%).

**Table 2:** Data sources and statistics for estimation of population attributable fractions (PAFs) in monozygotic twins.

Disease	Country	*N*_T_	*N*_C_	*N*_D_	P (%)	RR	PAF (%)	Ref.
Bladder cancer	Sweden, Denmark, Finland	15,668	5	189	5.03	8.28	4.42	[19]
Breast cancer (female)	Sweden, Denmark, Finland	8,437	42	505	14.3	4.60	11.2	[19]
Colorectal cancer	Sweden, Denmark, Finland	15,668	30	416	12.6	9.35	11.3	[19]
Leukemia	Sweden, Denmark, Finland	15,668	2	103	3.74	11.3	3.41	[19]
Lung cancer	Sweden, Denmark, Finland	15,668	18	296	10.8	11.4	9.89	[19]
Ovarian cancer (female)	Sweden, Denmark, Finland	8,437	3	125	4.58	6.13	3.83	[19]
Pancreatic cancer	Sweden, Denmark, Finland	15,668	3	123	4.65	11.8	4.26	[19]
Prostate cancer (male)	Sweden, Denmark, Finland	7,231	40	299	21.1	9.94	19.0	[19]
Stomach cancer	Sweden, Denmark, Finland	15,668	11	223	8.98	12.5	8.26	[19]
Thyroid autoimmunity	Denmark	284	7	17	45.2	14.3	42.0	[20]
Type 1 diabetes	Finland	4,307	3	20	23.1	99.1	22.8	[21]
Type 2 diabetes	Finland	4,307	29	113	33.9	25.3	32.6	[21]
Gallstone disease	Sweden	11,073	112	956	19.0	4.16	14.4	[22]
Alzheimer’s disease	Sweden	398	2	8	33.3	32.7	32.3	[23]
Dementia	Sweden	398	3	16	27.3	13.2	25.2	[23]
Chronic fatigue	Sweden	3,229	181	792	31.4	2.10	16.4	[24]
Gastroesophageal reflux disorder	Sweden	2178	95	370	33.9	3.48	24.2	[25]
Irritable bowel syndrome	Norway	1,252	14	97	22.4	5.49	18.3	[26]
Coronary heart disease death	Sweden	3,644	250	875	36.4	2.46	21.6	[27]
Stroke-related death	Denmark	3,852	35	316	18.1	4.20	13.8	[28]
General dystocia (female)	Sweden	928	40	173	31.6	2.93	20.8	[29]
Pelvic organ prolapse (female)	Sweden	3,376	34	157	30.2	12.6	27.8	[30]
Stress urinary incontinence (female)	Sweden	3,376	13	87	23.0	17.6	21.7	[30]
Migraine	European Union	9,077	382	1377	35.7	4.15	27.1	[31]
Rheumatoid arthritis	Finland	4,137	9	64	22.0	28.1	21.2	[32]
Asthma	Denmark	5,084	257	447	53.5	11.0	48.6	[33]
Parkinson disease	Sweden	8,590	9	151	10.6	12.0	9.76	[34]
Chronic obstructive pulmonary disease	Sweden, Denmark	7,747	18	149	19.5	20.0	18.5	[35]

Legend: *N*_T_, total twin pairs; *N*_C_, concordant twin pairs; *N*_D_, discordant twin pairs; P, proportion of case twins with an affected co-twin (%); RR, relative risk.

**Fig 1:**
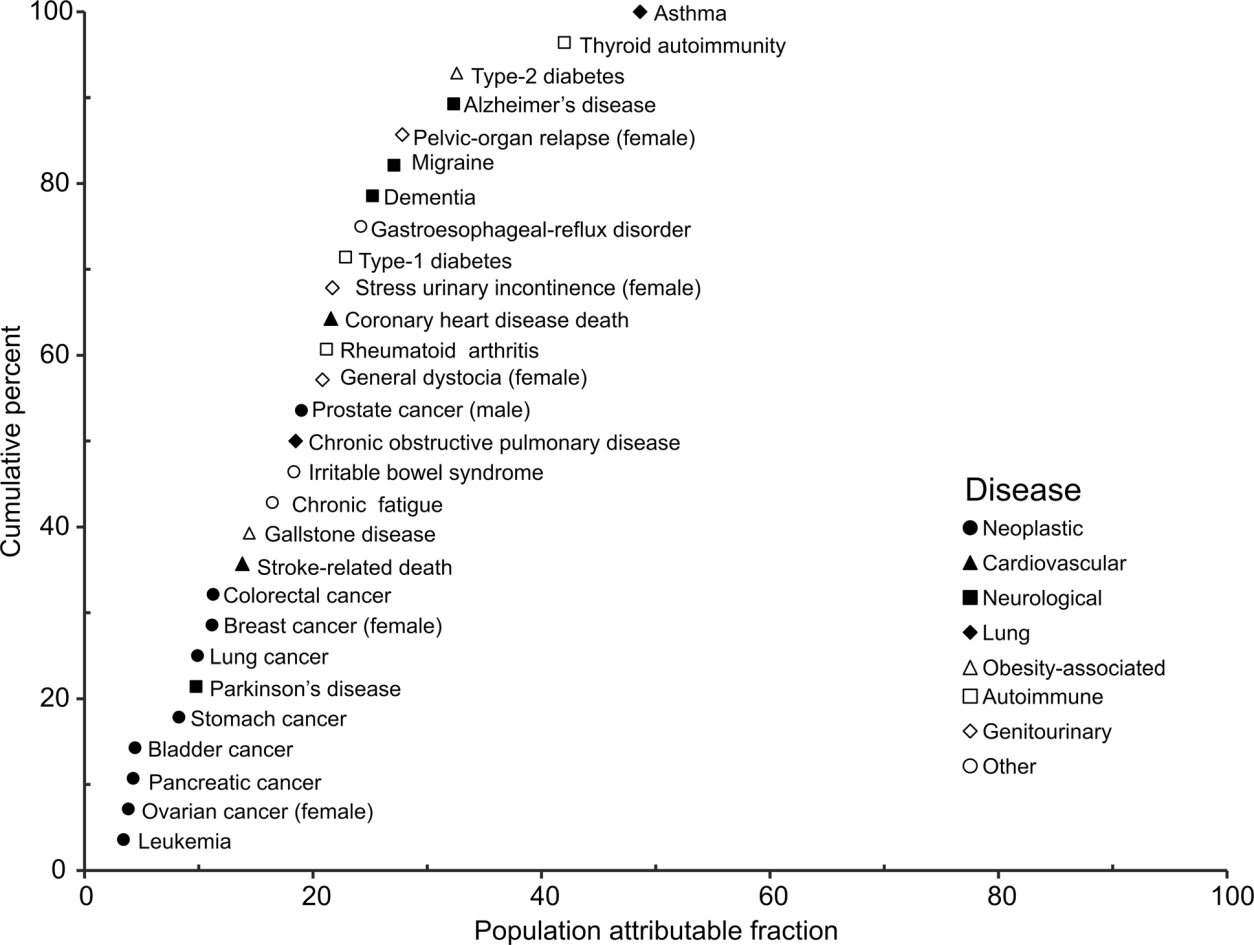
Population attributable fractions (PAFs) for 28 disease phenotypes estimated from studies of monozygotic twins. Sources of data and statistics are summarized in Table 2.

Fig 1 displays the cumulative distribution for the 28 phenotypes with symbols representing disease categories. Although there was variability within a given category, cancers tended to have the lowest PAFs (median = 8.26%) while neurological (median = 26.1%) and lung (median = 33.6%) diseases had the highest PAFs. Although these are apparently the first estimates of PAFs derived exclusively from MZ twins, Hemminki and Czene reported familial PAFs for cancers in the Swedish-Family Cancer Database (10.2 million individuals) [18] that are consistent with these results.

**Fig 2:**
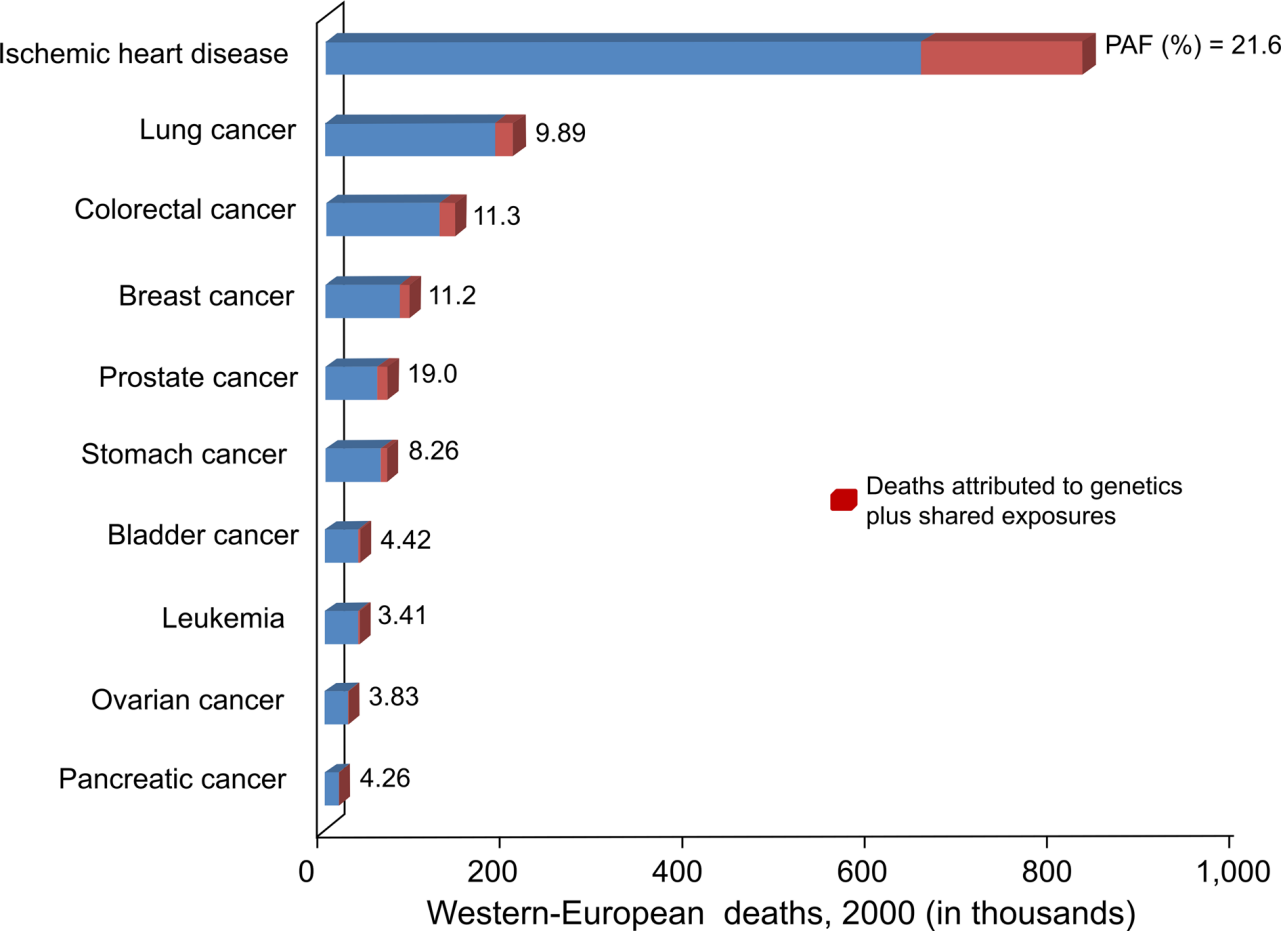
Numbers of Western-European deaths in 2000 estimated for ischemic heart disease and nine cancer types (1.53 million total deaths from these causes). The contributions attributed to genetics plus shared exposures are based on the population attributable fractions (PAFs) estimated from Western European monozygotic twins (Table 2).

Since heart disease and cancer are the two leading causes of mortality in Western Europe (and worldwide), the contributions of genetics plus shared exposures to incidence of these diseases were estimated as summarized in Fig 2. Assuming that the populations of MZ twins that were used to derive PAFs are reasonable surrogates for Western Europeans in the year 2000, then 0.25 million of the 1.53 million cancer and heart-disease deaths (16.4%) can be attributed to G-related factors.

Because comprehensive exposure data were not collected in the twin studies, it is not possible to directly estimate E-related PAFs or contributions to disease risks from G×E interactions. But given the modest values of G-related PAFs reported here, it is reasonable to infer that the combined effects of non-shared exposures (E) and G×E would be greater than those of G alone. This conjecture is supported by results of structural equation modeling by Lichtenstein *et al*. [19], who reported that non-shared exposures in monozygotic and dizygotic twins accounted for between 58% and 82% (median = 62%) of the variation in 12 types of cancer. Nonetheless, the hypothesized dominance of E and G×E on chronic-disease risks is at odds with a recent paper by Tomasetti and Vogelstein who found a strong correlation between cancer risks and total numbers of stem-cell divisions in various tissues, and concluded from this that ‘bad luck’ accounts for about two thirds of the variation in cancers [39]. In refuting this conclusion, Wu *et al*. pointed out that the bad-luck hypothesis is illogical because: it equates correlation with causation; it is inconsistent with the epidemiological evidence; and it requires that mutation signatures of cancers be correlated with age, which is rarely the case [15]. Recognizing that both intrinsic random errors and E-related factors can influence cancer risks, Wu *et al*. then applied models to the same data used by Tomasetti and Vogelstein [39], which allowed E-related factors to be estimated after adjustment for intrinsic random errors. Results indicated that E-related factors typically explained more than 90% of cancer risk, consistent with the small genetic PAFs observed for cancers in MZ twins (median = 8.26%).

## Conclusions

Because the human genome project planted the seeds for genome sequencing and large-scale omics technologies [5], it was inevitable that these methods would be used to search for causes of major diseases, and almost 2,000 GWAS have been reported [6]. Yet, the matrix of disease-associated genetic variants does not explain much heritability [7, 9]. Indeed, Yang *et al*. predicted that between 20 and 50 causal genetic variants would be required to explain half the burden of a common disease, depending on the frequency of each variant and risk ratio of the genotype [17]. The small genetic PAFs estimated here from studies of MZ twins (Table 2 and Fig 1) cast further doubt on the notion that our inherited genomes are the primary causes of chronic diseases. Nonetheless, the genome can influence disease outcomes through G×E interactions, and may also contribute through epistasis and heritable epigenetic effects that are as yet unknown. Thus, investigations of causes of chronic diseases should continue to consider genetic factors as part of a balanced strategy that characterizes both E and G with high resolution.

One avenue for discovering E-related risks would be to extend the data-driven approach embodied in GWAS and conduct exposome-wide association studies (EWAS) [40] via untargeted analyses of chemicals in blood (the ‘blood exposome’) [16]. Since disease processes can alter the blood exposome through dysregulation of systems biology, it is important that EWAS be conducted with archived biospecimens collected prior to diagnosis from incident cases and matched controls in prospective cohort studies. This makes it possible to distinguish chemical signatures of potentially causal exposures from those generated by progression of the disease (reverse causality) [40, 41].

A good example of this data-driven approach for EWAS is given by Wang *et al*. [42] who found 18 chemical features (out of more than 2000 detected) that were associated with cardiovascular disease in samples totaling only 75 incident cases and 75 matched controls. Three of the features were identified as choline and its metabolites, betaine, and trimethylamine-*N*-oxide (TMAO), with TMAO exhibiting the strongest disease risk in follow-up studies [43, 44]. Since TMAO is a product of joint microbial and human metabolism of choline, the positive association between plasma TMAO and disease risk points to possible involvement of the gut microbiota in the etiology of cardiovascular disease. It is interesting that a study of colorectal cancer by Bae *et al*. [45] also found a positive association between plasma TMAO and disease risk, again suggesting involvement of the gut microbiota.
